# Reproductive Efficiency of a Mediterranean Endemic Zooxanthellate Coral Decreases with Increasing Temperature along a Wide Latitudinal Gradient

**DOI:** 10.1371/journal.pone.0091792

**Published:** 2014-03-11

**Authors:** Valentina Airi, Francesca Gizzi, Giuseppe Falini, Oren Levy, Zvy Dubinsky, Stefano Goffredo

**Affiliations:** 1 Marine Science Group, Department of Biological, Geological and Environmental Sciences, Section of Biology, Alma Mater Studiorum – University of Bologna, Bologna, Italy, European Union; 2 Department of Chemistry “G. Ciamician,” Alma Mater Studiorum – University of Bologna, Bologna, Italy, European Union; 3 The Mina and Everard Goodman Faculty of Life Sciences, Bar-Ilan University, Ramat-Gan, Israel; U.S. Geological Survey, United States of America

## Abstract

Investments at the organismal level towards reproduction and growth are often used as indicators of health. Understanding how such energy allocation varies with environmental conditions may, therefore, aid in predicting possible responses to global climatic change in the near future. For example, variations in seawater temperature may alter the physiological functioning, behavior, reproductive output and demographic traits (e.g., productivity) of marine organisms, leading to shifts in the structure, spatial range, and abundance of populations. This study investigated variations in reproductive output associated with local seawater temperature along a wide latitudinal gradient on the western Italian coast, in the zooxanthellate Mediterranean coral, *Balanophyllia europaea*. Reproductive potential varied significantly among sites, where *B. europaea* individuals from the warmest site experienced loss of oocytes during gametogenesis. Most of the early oocytes from warmest sites did not reach maturity, possibly due to inhibition of metabolic processes at high temperatures, causing *B. europaea* to reabsorb the oocytes and utilize them as energy for other vital functions. In a progressively warming Mediterranean, the efficiency of the energy invested in reproduction could be considerably reduced in this species, thereby affecting vital processes. Given the projected increase in seawater temperature as a consequence of global climate change, the present study adds evidence to the threats posed by high temperatures to the survival of *B. europaea* in the next decades.

## Introduction

Coral reefs, like many other ecosystems, are currently undergoing changes in biodiversity, ecosystem function, and resilience due to rising seawater temperatures acting in synergy with additional environmental pressures [1]. A rise in global average temperature of 0.7°C since the start of the industrial revolution has caused or contributed to significant losses of global coral cover over the past few decades, and oceans are expected to experience a further warming of 1.1–6.4°C within the 21^st^ century [2]. Climatic models [3] predict that the Mediterranean basin will be one of the most impacted regions by the ongoing warming trend [4]. The Mediterranean is already showing rates of seawater warming that exceed threefold those of the global ocean [2], [4], making it a potential model for global scenarios to occur in the world's marine biota, and a natural focus of interest for research [5].

Increasing temperatures are having a strong impact on marine systems [6]. Indeed, temperature is the major environmental factor controlling invertebrate development, marine species distributions and recruitment dynamics [7], [8]. Seawater temperature increases will likely affect the population biology of coral species by reducing reproductive capacity [9]. The harmful effects of increasing temperature on coral reproduction include reduced individual fecundity, egg quality, lowered fertilization success and reduced recruitment through effects on post-fertilization processes (e.g., embryonic development, larval development, survival, settlement, metamorphosis, and early post-settlement growth) [10], [11]. The combined effects of fertilization failure and reduced embryonic development in some coral species are likely to exacerbate ecological impacts of climate change by reducing biodiversity [12]. Several studies assessed the immediate and delayed impacts of environmental change on Mediterranean gorgonian colonies [11]–[14] including sublethal impacts on reproductive effort [11], [15], [16], [17], but few studies have examined temperate solitary corals. Research focusing on reproductive processes in regions with peculiar physical conditions is urgently needed as a baseline against which to test the effects of climate change on sexual reproduction (e.g. fecundity) [10], [18] and organismal performance, that are essential to understand population dynamics of marine organisms [19].

Organismal performance under both “normal” and “stressful” conditions is mainly determined by the energetic status of the individual, which can ultimately affect its fitness (i.e. reproductive output). During prolonged periods of stress, the energy balance of a coral is negative and the organism is drawing on all biochemical pools, and thus both storage and structural components for energy could be compromised [20]. Shallow water reef corals strongly rely on energy derived from photosynthesis by its symbiotic zooxanthellae [21]. In particular, key processes like gametogenesis [22], larval longevity and settlement [23] are dependent on the availability of stored energy as lipids that are reabsorbed when resources are limited [24]. If metabolic processes involved in recovery from stress deplete lipid reservoirs in oocytes, then fewer resources are available for new egg production [25], significantly affecting gametogenesis.

This study focused on an endemic zooxanthellate Mediterranean scleractinian, *Balanophyllia europaea* (Fig. S1), a simultaneous hermaphrodite and brooding coral [26]. There is growing concern for the future of this endemic species in light of expected seawater warming, since increasing temperature negatively affects *B. europaea* skeletal density [27] (due to increased porosity [28]), population abundance [29], population structure stability [30], growth and calcification [28]. Our specific aim was to quantify the reproductive output of *B. europaea* along a latitudinal gradient of temperature. We expected to find a similar negative response of reproductive output with increasing temperature.

## Materials and Methods

### Ethics Statement

This study was carried out following the fundamental ethical principles. According to the European normative, there is no active conservation measure for the Mediterranean scleractinian coral studied here (*B. europaea*). The species is not protected in Italy, nor is it subject to any regulations. Thus, no permit was needed to sample specimens. For this study, sampling was limited strictly to the number necessary and performed where the species has high population density to minimize the impact of removing individuals and preserve both the demographic and genetic structure of the natural populations.

**Figure 1:**
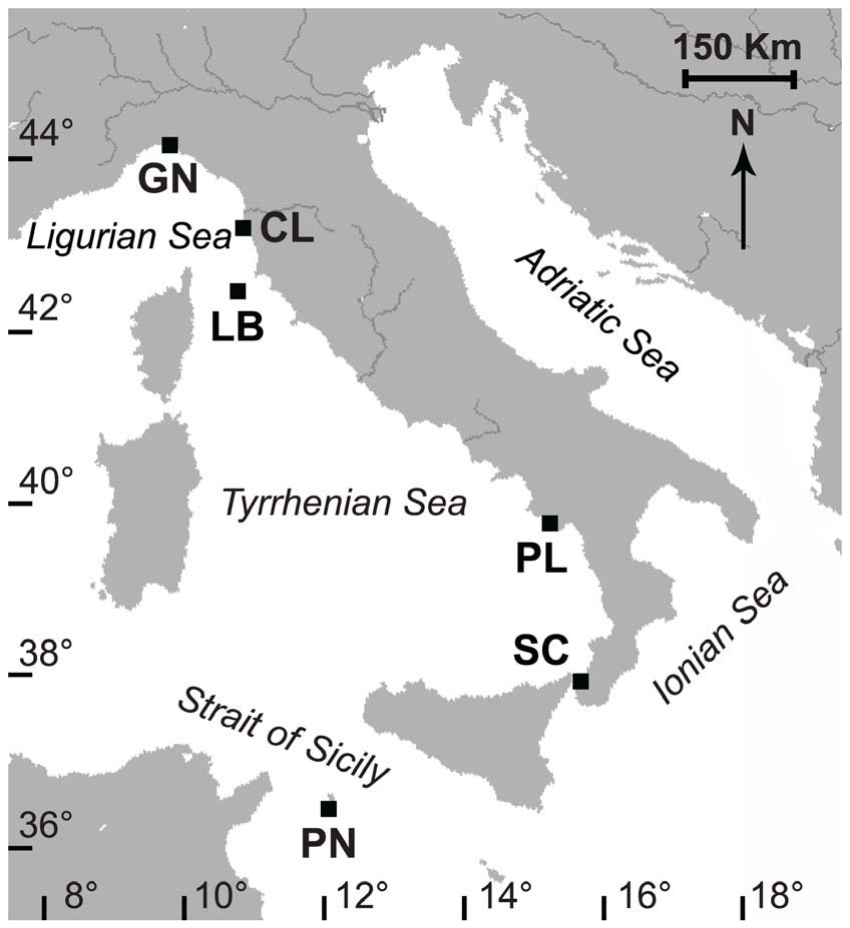
Map of the Italian coastline indicating the sites where corals were collected. Abbreviations and coordinates of the sites in decreasing order of latitude: GN Genova, 44°20′N, 9°08′E; CL Calafuria, 43°27′N, 10°21′E; LB Elba Isle, 42°45′N, 10°24′E; PL Palinuro, 40°02′N, 15°16′E; SC Scilla, 38°01′N, 15°38′E; PN Pantelleria Isle, 36°45′N, 11°57′E.

Specimens of *B. europaea* came from six sites along a latitudinal gradient, from 44°20′N to 36°45′N (Fig. 1). Coral collection began in June 2010 and ended in November 2012. During this period, 18 samples were taken monthly from five populations (Genova: April 2011-September 2012; Elba: December 2010-May 2012; Palinuro: June 2010-November 2011; Scilla: June 2011-November 2012; Pantelleria: June 2011-November 2012), with a minimum of 15 polyps collected during each excursion. Data from Calafuria population came from a previous study [26] in which samples were collected from July 1997 to October 1998.

Biometric analyses were performed by measuring length (L, maximum axis of the oral disc), width (W, minimum axis of the oral disc) and height (h, oral–aboral axis) of each sampled polyp. The volume (V) of the individual polyp was calculated using the formula [26].

Polyps were post-fixed in Bouin solution. After decalcification in EDTA and dehydration in a graded alcohol series from 80% to 100%, polyps were embedded in paraffin and serial transverse sections were cut at 7 μm intervals along the oral-aboral axis, from the oral to the aboral poles. Tissues were then stained with Mayer's haematoxylin and eosin [26].

Cytometric analyses were made with an optical microscope using the image analyzer NIKON NIS-Elements D 3.2. The maximum and minimum diameters of oocytes in nucleated sections and spermaries were measured and the presence of embryos in the coelenteric cavity was recorded. Spermaries were classified into five developmental stages in accordance with earlier studies on gametogenesis in scleractinians [19], [31], [32].

Reproductive output was defined through three reproductive parameters: a) *fecundity rate* and *spermary abundance*, both defined as the number of reproductive elements per body volume unit (100 mm^3^); b) *“gonadal” index*, defined as the percentage of body volume occupied by germ cells [26]); and c) *reproductive element size*, defined as the average of the maximum and minimum diameter of spermaries and oocytes in nucleated section [26].

Based on the reproductive season [26], gametal development in *B. europaea* was divided in two gamete activity periods. The *gametes recruitment period* [33], [34] was defined as the post-fertilization period, between June and September, generally characterized by: 1) a stock of smaller oocytes; 2) the recruitment of new oocytes; and 3) the beginning of spermary development [26]. The *gametes maturity period* [33], [34] was defined as the pre-fertilization period taking place between December and March and generally characterized by the presence of larger oocytes and advanced stage of maturation of spermaries [26].

Temperature data (Depth Temperature – DT; °C) came from temperature sensors (I-Button DS1921H, Maxim Integrated Products), placed at the sampling location for each population. Sensors recorded temperatures during the entire experimental period. Sea Surface Temperature data (SST; °C) for each site were recorded hourly from the National Mareographic Network of the Institute for the Environmental Protection and Research (ISPRA, available to http://www.mareografico.it). These data are measured by mareographic stations placed close to the sampling sites using SM3810 manufactured by the Society for the Environmental and Industrial monitoring (SIAP+MICROS). A linear regression was produced between DT and SST data to estimate historical at-depth temperatures. In this study we considered the average DT temperature of the three years preceding the sampling (n = 36 monthly temperatures).

Solar radiation (W/m^2^) was collected from the archives of the Satellite Application Facility on Climate Monitoring (CM-SAF/EUMETSAT, available to http://www.cmsaf.eu), using real time data sets based on intersensor calibrated radiances from MFG satellites. Mean annual solar radiation of each site was obtained for the 2.5°-latitude-by-longitude square associated with each of the six sites. As for temperature, also for solar radiation we considered the average of the three years preceding the sampling (n = 36 monthly solar radiation).

Data were checked for normality using a Kolmogorov-Smirnov's test and for variance homoskedasticity using a Levene's test. When assumptions for parametric statistics were not fulfilled, a nonparametric test was used. The Kruskal–Wallis test is a non-parametric alternative to the analysis of variance (ANOVA) and is used to compare groups of means; it is useful for data that do not meet ANOVA's assumptions. The non-parametric Kruskal–Wallis test was used to compare reproductive parameters among study sites. The non-parametric Kolmogorov-Smirnov test was used to compare the size-frequency distribution of reproductive elements between populations and between the two periods. Student's *t* test was used to compare the mean oocytes and spermaries size of populations between periods. Spearman's rank correlation coefficient was used to calculate the significance of the correlations between reproductive and environmental parameters. Spearman's rank correlation coefficient is an alternative to Pearson's correlation coefficient [35]. It is useful for data that are non-normally distributed and do not meet the assumptions of Pearson's correlation coefficient [36]. All analyses were computed using PASW Statistics 17.0.

## Results

Mean annual solar radiation (W/m^2^) and mean annual DT (°C) were significantly different among sites (solar radiation, ANOVA, p<0.001; DT, Kruskal-Wallis, p<0.05; Table 1; Fig S2).

**Table 1:** Mean annual solar radiation (W/m^2^) and temperature (DT; °C) values of the sampled populations.

Population	Code	DT (°C) mean ± SE	Solar radiation (W/m^2^) mean ± SE
Calafuria	CL	17.73±0.16	174.1±1.9
Elba	LB	18.07±0.24	184.9±2.3
Genova	GN	18.13±0.43	156.9±3.2
Scilla	SC	18.73±0.15	205.5±1.8
Palinuro	PL	19.14±0.14	194.6±2.7
Pantelleria	PN	19.69±0.05	218.2±0.5

DT sensors (I-Button DS1921H, Maxim Integrated Products), were placed at the sampling location, at 5–7 m depth in each population. Solar radiation (W/m^2^) was collected from MFG satellites. The sites are arranged in order of increasing DT; SE, standard error.

**Figure 2:**
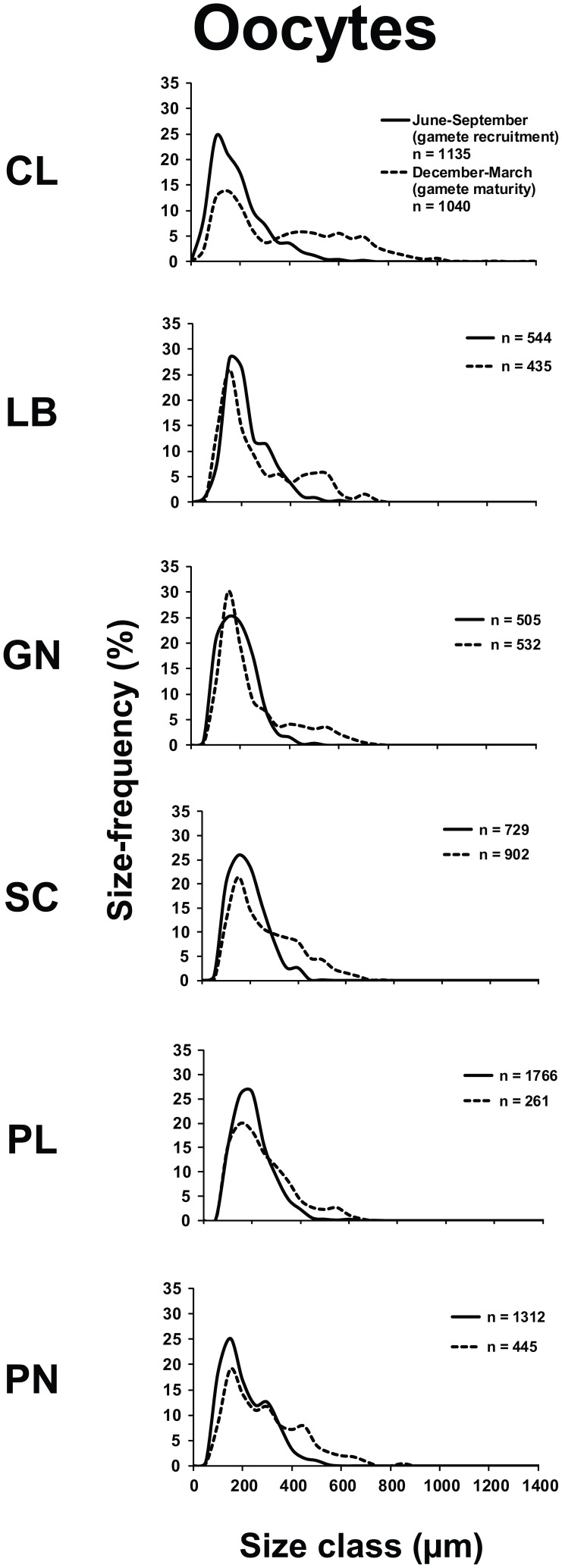
Oocyte size/frequency distribution in the recruitment and maturity periods. Distribution of the oocytes size during gamete recruitment period (solid line) and gamete maturity period (dashed line).

All populations contained both oocytes and spermaries during both reproductive periods, while embryos were detected only between June and September (gametes recruitment period). The oocyte size/frequency distribution of June-September (gametes recruitment period) was significantly different from that of December-March (gametes maturity period), in all populations (Kolmogorov-Smirnov, p<0.001; Fig. 2). Within June and September (gametes recruitment period) most oocytes were smaller than 400 μm, in all populations. In the following season (December-March, gametes maturity period), two distinct oocyte stocks appeared in all populations, characterized respectively by small (immature<400 μm) and large (mature >400 μm) cells (Fig. 2). The mean oocyte size of June-September (gametes recruitment period) was significantly lower than that of December-March (gametes maturity period) in all populations (Student's *t*-test, p<0.001; Table 2; Fig. S3).

**Table 2:** Mean fecundity, gonadal index and diameter of oocytes in each population.

Gametes recruitment period (June – September)					
Population	N	Fecundity (#/100 mm^3^) mean ± SE	Gonadal Index (%) mean ± SE	N	Diameter (μm) mean ± SE
Calafuria	18	161±39	0.22±0.07	1135	166.3±3.3
Elba	6	148±37	0.65±0.17	544	193.7±3.8
Genova	8	168±47	0.27±0.12	505	166.0±3.3
Scilla	9	256±58	0.41±0.13	729	166.7±2.8
Palinuro	10	734±194	1.57±0.38	1766	178.4±1.9
Pantelleria	8	663±240	1.43±0.51	1312	188.2±2.6

Gametes maturity period (December – March)					
Population	N	Fecundity (#/100 mm^3^) mean ± SE	Gonadal Index (%) mean ± SE	N	Diameter (μm) mean ± SE
Calafuria	19	117±38	1.04±0.30	1040	350.3±7.5
Elba	8	175±32	0.79±0.16	435	243.4±7.7
Genova	4	411±183	1.37±0.40	532	222.5±6.2
Scilla	4	602±257	2.72±1.50	902	241.1±4.5
Palinuro	7	112±30	0.39±0.15	261	217.7±7.5
Pantelleria	6	236±106	1.25±0.41	445	265.4±7.1

Mean fecundity, gonadal index and diameter of oocytes in each population for both reproductive periods. The sites are arranged in order of increasing DT; SE, standard error. N, polyp number for fecundity and gonadal index, oocyte number for diameter.

**Figure 3:**
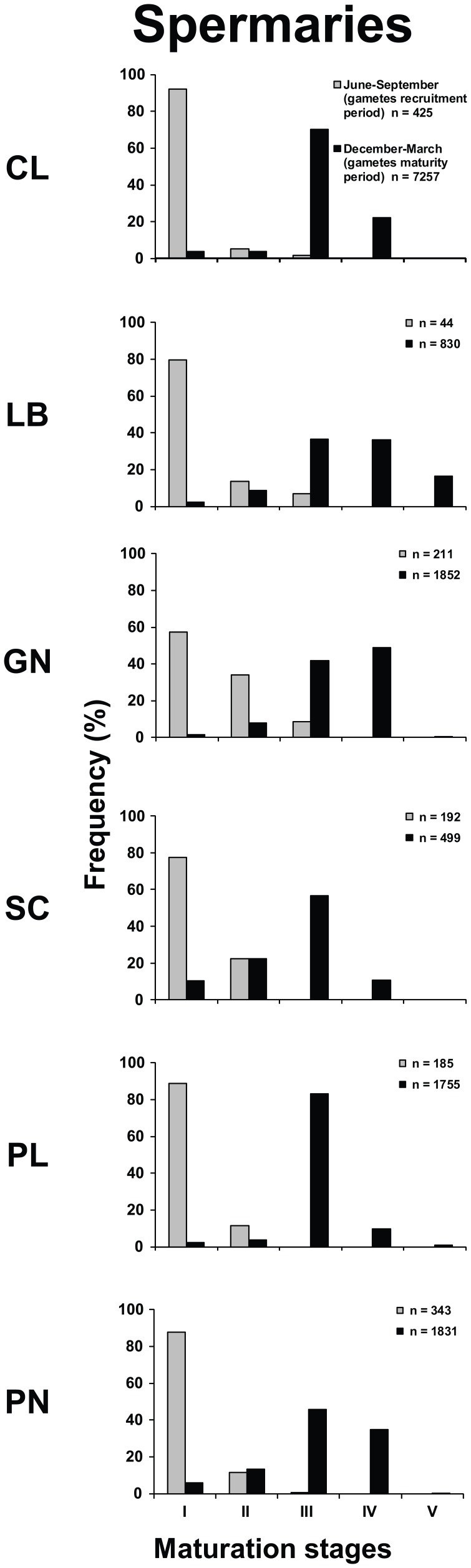
Spermary frequency distribution in the recruitment and maturity periods. Distribution of the maturation stages during gamete recruitment period (gray histogram bars) and gamete maturity period (black histogram bars).

The distribution of spermary maturation stages in June-September (gametes recruitment period) was significantly different from that in December-March (gametes maturity period), in all populations (Kolmogorov-Smirnov, p<0.001; Fig. 3). Each population was characterized, from June to September (gametes recruitment period), by small spermaries, mainly belonging to the earliest maturation stages (stages I and II). In the period December-March (gametes maturity period), all populations were characterized by more advanced maturation stages (mainly stage III; Fig. 3). The mean spermary size of June-September (gametes recruitment period) was significantly lower than that of December-March (gametes maturity period) in all populations (Student's *t*-test, p<0.001; Table 3; Fig. 3). In all populations, June-September (gametes recruitment period) was characterized by the presence of embryos in the coelenteric cavity.

**Table 3:** Mean abundance, gonadal index and diameter of spermaries in each population.

Gametes recruitment period (June – September)					
Population	N	Abundance (#/100 mm^3^) mean ± SE	Gonadal Index (%) mean ± SE	N	Diameter (μm) mean ± SE
Calafuria	17	140±52	0.010±0.003	425	51.4±1.2
Elba	2	169±106	0.010±0.001	44	54.2±2.8
Genova	1	1463	0.080	211	46.3±1.1
Scilla	6	272±80	0.010±0.004	192	40.7±0.8
Palinuro	6	393±133	0.020±0.006	185	40.0±1.0
Pantelleria	5	760±368	0.030±0.020	343	42.0±0.7

Gametes maturity period (December – March)					
Population	N	Abundance (#/100 mm^3^) mean ± SE	Gonadal Index (%) mean ± SE	N	Diameter (μm) mean ± SE
Calafuria	19	1840±609	1.10±0.40	7257	120.5 ±0.8
Elba	8	595±235	0.47±0.23	830	126.0 ±1.8
Genova	4	2135±1122	1.95±1.51	1852	124.8 ±1.3
Scilla	4	981±561	0.16±0.09	499	81.7 ±1.6
Palinuro	6	1875±1664	0.85±0.80	1755	103.2 ±1.1
Pantelleria	5	2660±2320	0.93±0.25	1831	92.0 ±1.0

Mean abundance, gonadal index and diameter of spermaries in each population for both reproductive periods. The sites are arranged in order of increasing DT; SE, standard error. N, polyps number for abundance and gonadal index, spermaries number for diameter.

Fecundity, gonadal index and oocyte size were significantly different among populations, during June-September (gametes recruitment period) (fecundity, Kruskal–Wallis test, p<0.01; gonadal index and oocyte size, Kruskal–Wallis test, p<0.001; Tables 2 and S1). In this period, all oocyte reproductive parameters showed positive correlations with both environmental parameters (DT and solar radiation; Table S1; Fig. S4). During December-March (gametes maturity period), the fecundity and oocyte size were significantly different among populations (fecundity, Kruskal–Wallis test, p<0.05; diameter, Kruskal–Wallis test, p<0.001; Tables 2 and S1). The mean size of oocytes across all populations was negatively correlated with the DT (Table S1; Fig. S5). In the warmest population (Pantelleria island, 19.69±0.05°C; Table 1), the number of mature oocytes at fertilization was three times lower than in the recruitment period, indicating a clear reduction of fecundity during this period (Table 2). In the coldest population (Calafuria, 17.73±0.16°C; Table 1), fecundity was the same during both periods (Table 2).

In both periods, only the spermary size was significantly different among populations (Kruskal–Wallis test, p<0.001; Tables 3 and S2) and in both reproductive periods, spermary size was negatively correlated with both DT and solar radiation (Table S2; Fig. S6 and S7).

## Discussion

Traditionally, seawater temperature cycles and solar radiation fluctuations have been related to reproductive timing of gamete development, fertilization and planulation [16], [37] providing a reliable cue to reset the biological clock and trigger the physiological changes related to oocyte yolk deposition [38] and spermary development [26], [39], [40]. The effects of changing photoperiod and seawater temperature on gametogenic cycles of anthozoans have been largely overlooked [15], [41], [42]. The reproductive biology of *B. europaea*, studied at Calafuria, shows a reproductive seasonality induced by annual variation of seawater temperature and photoperiod [26]. The same pattern seems to appear in other Mediterranean dendrophylliids like *Leptopsammia pruvoti* [39] and *Astroides calycularis* [40] and in the Mediterranean endemic oculinid *Cladocora caespitosa* [43], [44]. A similar periodicity for gamete development and embryonic presence during the recruitment period, suggest an overlap of reproductive seasonality in all populations along the latitudinal gradient by *B. europaea*. In broadcasting scleractinian corals, where temperature dependence leads to location-specific synchronous reproductive times [45], temporal variation in spawning events by corals from different latitudes, over two or more consecutive months, is uncommon [18]. In brooding scleractinians, reproductive cycles are protracted over several months coinciding with environmental seasonality change [46], [47].

Specimens from the warmer and more irradiated populations of *B. europaea* generated a significantly greater number of oocytes during the initial stages of gametogenesis (gametes recruitment period). Before fertilization (gametes maturity period), however, individual oocyte number was not related to temperature/irradiance along the gradient, while oocyte size was smaller with increasing temperature (Tables 2 and S1). A reduction of photosynthetic efficiency is documented for several species when temperatures are above optimal [48], [49], thereby limiting energetic resources for polyp gametogenesis [9], [50]. The onset of gametogenesis (proliferation of germ cells and their differentiation into gametes) may require little energy investment and may, therefore, be less sensitive to selective pressures such as food availability and more reliant on environmental seasonal cycles [51]. In this scenario, warmer populations of *B. europaea* could invest in energetically inexpensive early stages of oogenesis to generate a potential energy resource that would guarantee sufficient metabolic efficiency. On the other hand, the ripening of gametes, especially of oocytes, is an energy consuming process and, therefore, extremely sensitive to selective pressures [51].

Regarding male gametogenesis, during both reproductive periods, the size of spermaries decreased with increasing temperature (Tables 3, S2), while their abundance was not significantly related to environmental parameters. The energetic investment for gametogenesis between males and females is often assumed to differ [52]. For many lower invertebrates, and especially sessile ones, mating effort and parental care are minimal and reproductive output provides a good approximation of the reproductive effort, so most of the energy involved in reproduction is stored in gonads [53]. This “cost of sex” is mainly represented by oogenesis, while the investment of spermary production minimally influences the energetic balance of the organism [52].

For all organisms, energy flow provides an important cost for physiological performance, including maintenance, growth and reproduction, all of which have implications on survival and fitness. Reproductive investment and growth are often used as indicators of health or stress at the organism level (e.g. [54]), and knowledge of how such allocation varies among species or morphological types is crucial for the interpretation of physiological response to environmental factors [53]. Essentially, organisms invest their energy in continuous trade-offs between somatic/skeletal growth and reproduction, which in many species includes the possibility of asexual reproduction [55]. In a changing environment, physiological trade-offs vary through time, reflecting variations in resource availability [56], and the ‘energy allocation’ explains this partitioning between the various investment options (e.g. growth, sexual reproduction, defense) [57]. For example, the coral *Montipora digitata* under varying light regimes shows an increase of energy allocated to reproduction versus growth at intermediate light levels. In this species the skeletal growth is less susceptible to environmental variations and during periods of resource shortage, energy is preferentially allocated for skeletal growth [57]. *B. europaea* shows a reduction of skeletal density, due to increasing porosity, and especially of pores with larger size, with increasing temperature [28], [29], [58]. Also its growth and calcification are negatively related to temperature [27], [30]. Warmer populations are less stable, showing a progressive reduction in young individuals and reduced population density [29], [30]. It has been hypothesized that the decrease in calcification rate [27] and skeletal density [29] in *B. europaea* with increasing temperature could be due to a reduction of energy input available, maybe due to photosynthetic inhibition of the symbionts [29], [30]. Populations of *B. europaea* in warmer sites could potentially resorb earlier oocytes adjusting their energetic budget by reallocating the resources destined to oocyte maturity into other vital functions depleted by the negative effect of temperature. Resorption of oocytes is not fully understood, but it is thought that by breaking down the large amount of lipid vesicles in oocytes, energy can be absorbed back into the coral [59]. In the soft coral *Lobophytum compactum*, fecundity is reduced after an induced bleaching event. In this zooxanthellate coral, early oocytes are resorbed to allow development of remaining ones. Energy allocated to reproduction is apparently shifted towards maintaining fewer eggs than normal to ensure that they reach a mature size [37]. The branching coral *Acropora formosa* shows lower survival rate and a resorption of early vitellogenic oocytes after fragmentation, suggesting that there is a trade-off of energy between reproduction and survival [60].

In conclusion, *B. europaea* shows the highest ecological performance in the coldest part of its distribution, characterized by a higher growth coefficient [30], a greater population density [29], [61] and a higher efficiency in partitioning the energy budget (this work; [27]-[30]). On the contrary, populations in warmer regions appear to invest their energy in the initial stages of gametogenesis in order to ensure a sufficient gamete number ready for fertilization in the maturity period. Nevertheless, this effort is not enough to guarantee the same reproductive performance at higher temperatures, as adult populations in warmer sites are less abundant, less stable, and contain fewer young individuals [29], [30]. This suggests that increasing temperature may negatively influence post-fertilization life stages, such as larval dispersal, survival and settlement. Depressed organismal condition exhibited by the warmer population could be due to their location near the edge of the species distribution range, where species generally show a lower ecological performance with reduced adaptability to variations in climate [62]. Being endemic to the Mediterranean [63], *B. europaea* has limited potential to respond to seawater warming by migrating northward toward lower temperatures, since the latitudinal range considered covers almost the entire northern distribution of this species [27]. This scenario would indicate a possible reduction in the distribution area of this species, with irrecoverable losses in terms of genetic variability, particularly considering the fragmented genetic structure that characterizes the species [64]. The present study, therefore, confirms the concerns for the future of this endemic species [27]–[30]. In fact, in a progressively warming Mediterranean, the energetic efficiency of this species could be considerably reduced, affecting vital processes (e.g. growth). Thus, an effective allocation strategy will be crucial for ensuring adaptability to a changing environment.

## Supporting Information

Figure S1 **Living specimens of** ***Balanophyllia europaea*** **photographed at Scilla (South Italy, 38°01′N, 15°38′E)**.
(TIF)

Figure S2 **Annual fluctuation of solar radiation and temperature.** Mean monthly solar radiation (W/m^2^) and temperature (DT; °C) during three years preceding the sampling. Annual fluctuation referred to January 1995 - December 1997 in the Calafuria population. For the other five populations it referred to January 2009 - December 2011.
(EPS)

Figure S3 **Oocyte diameter during recruitment and maturity periods.** Monthly size increase of the oocyte diameter during gamete recruitment (gray indicators) and maturity (black indicators) period.
(EPS)

Figure S4 **Oocytes. Correlation analyses.** Spearman's correlation between reproductive and environmental parameters during gamete recruitment period; N, polyp number for fecundity and gonadal index, oocyte number for diameter; r_s_, Spearman's correlation coefficient; p, significance of the correlation test.
(EPS)

Figure S5 **Oocytes. Correlation analyses.** Spearman's correlation between reproductive and environmental parameters during gamete maturity period; N, polyp number for fecundity and gonadal index, oocyte number for diameter; r_s_, Spearman's correlation coefficient; p, significance of the correlation test.
(EPS)

Figure S6 **Spermaries. Correlation analyses.** Spearman's correlation between reproductive and environmental parameters during gamete recruitment period; N, polyps number for abundance and gonadal index, spermaries number for diameter; r_s_, Spearman's correlation coefficient; p, significance of the correlation test.
(EPS)

Figure S7 **Spermaries. Correlation analyses.** Spearman's correlation between reproductive and environmental parameters during gamete maturity period; N, polyps number for abundance and gonadal index, spermaries number for diameter; r_s_, Spearman's correlation coefficient; p, significance of the correlation test.
(TIF)

Table S1 **Oocytes.** Kruskal-Wallis test and correlation analyses between reproductive and environmental parameters in the sampled populations, in both periods.
(DOC)

Table S2 **Spermaries.** Kruskal-Wallis test and correlation analyses between reproductive and environmental parameters in the sampled populations, in both periods.
(DOC)
