# Supplementary figures and images for: Reproductive Efficiency of a Mediterranean Endemic Zooxanthellate Coral Decreases with Increasing Temperature along a Wide Latitudinal Gradient
Source: PLoS One. 2014 Mar 11;9(3):e91792. doi: 10.1371/journal.pone.0091792 (PMC3950289; doi:10.1371/journal.pone.0091792)

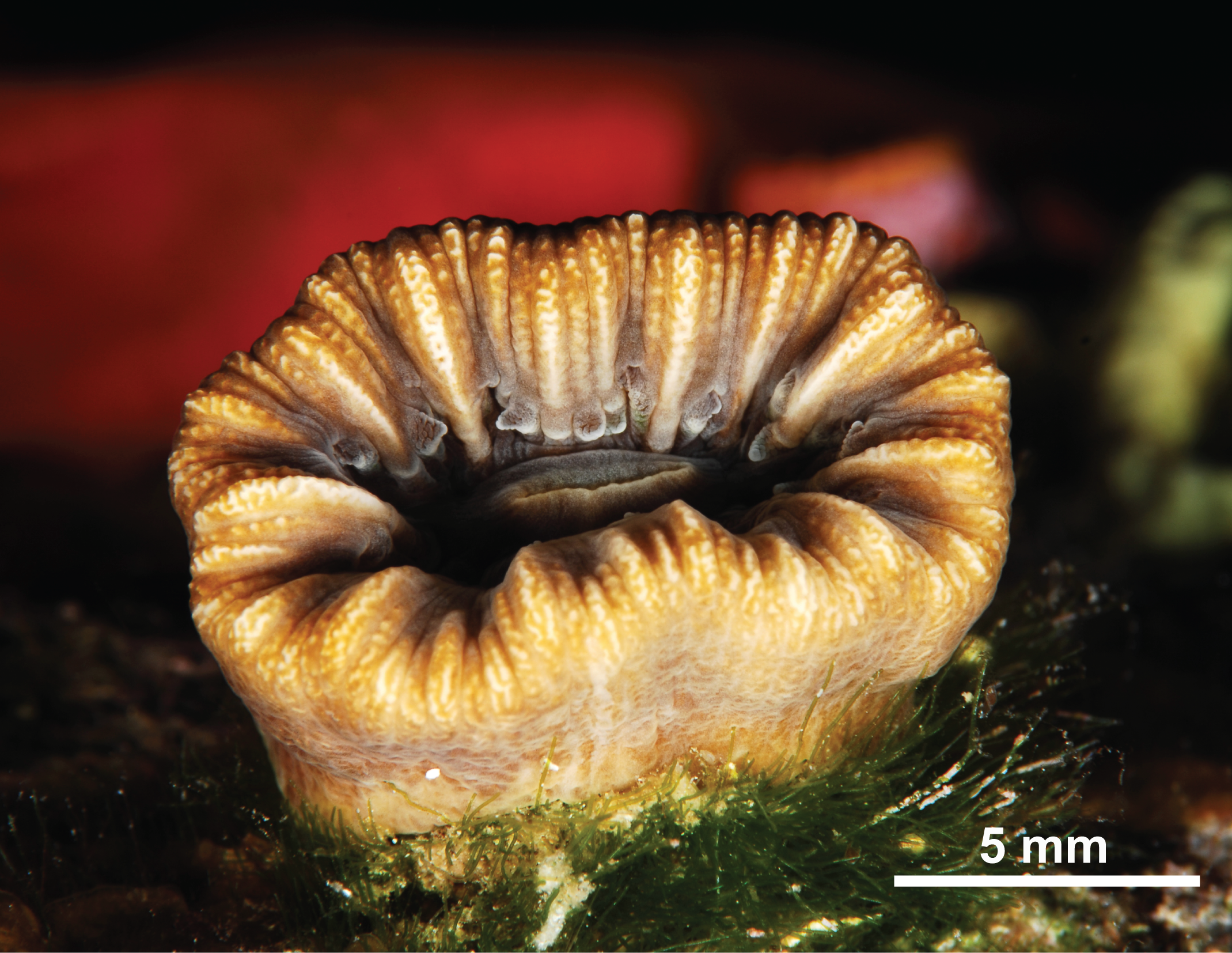

Supplement: Figure S1 — Living specimens of Balanophyllia europaea photographed at Scilla (South Italy, 38°01′N, 15°38′E). (TIF) [file pone.0091792.s001.tif]

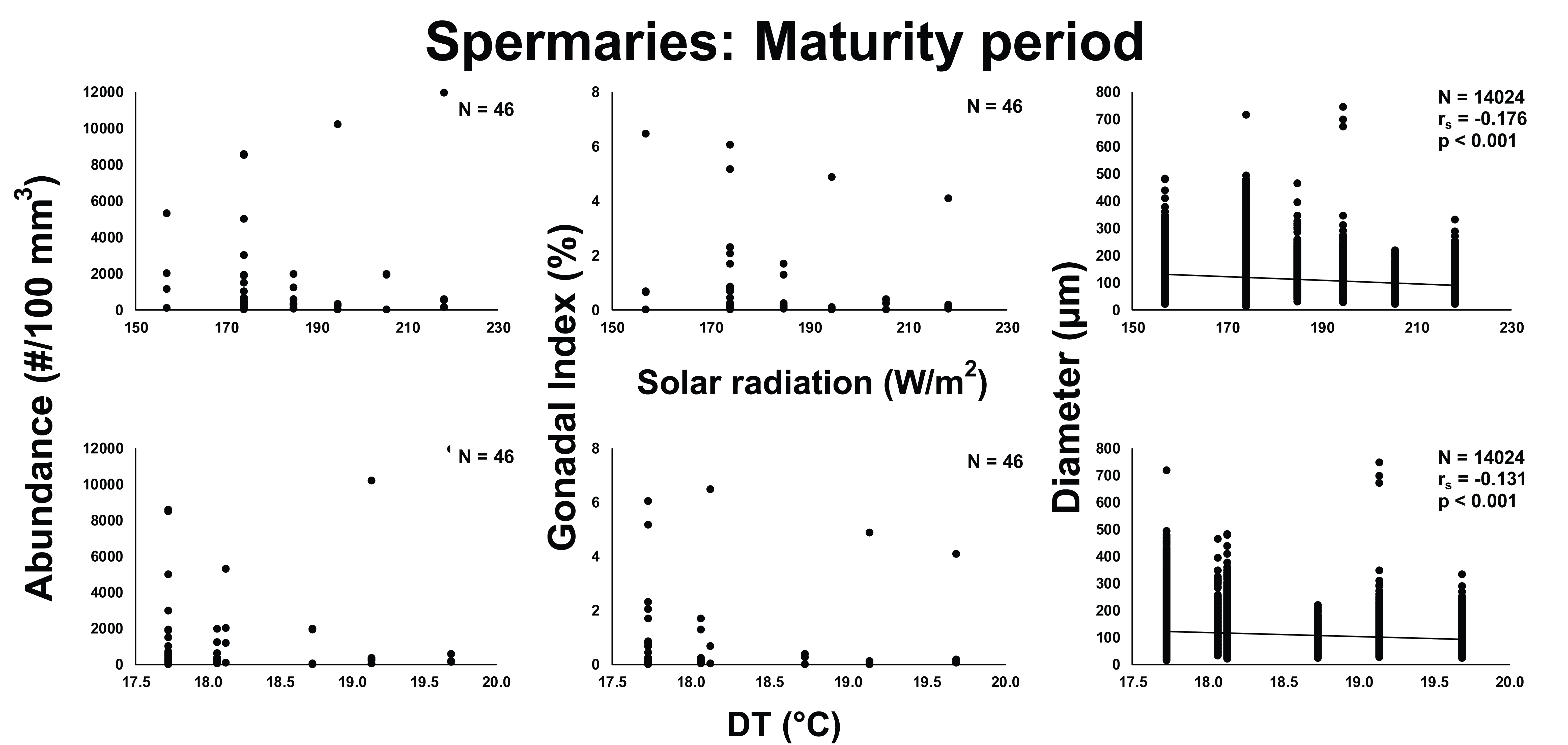

Supplement: Figure S7 — Spermaries. Correlation analyses. Spearman's correlation between reproductive and environmental parameters during gamete maturity period; N, polyps number for abundance and gonadal index, spermaries number for diameter; rs, Spearman's correlation coefficient; p, significance of the correlation test. (TIF) [file pone.0091792.s007.tif]
