# Supplementary material for: Reproductive Efficiency of a Mediterranean Endemic Zooxanthellate Coral Decreases with Increasing Temperature along a Wide Latitudinal Gradient
Source: PLoS One. 2014 Mar 11;9(3):e91792. doi: 10.1371/journal.pone.0091792 (PMC3950289; doi:10.1371/journal.pone.0091792)
Supplement: Table S1 — Oocytes. Kruskal-Wallis test and correlation analyses between reproductive and environmental parameters in the sampled populations, in both periods. (DOC) [file pone.0091792.s008.doc]

**Table S1.** Oocytes. Kruskal-Wallis test and correlation analyses between reproductive and environmental parameters in the sampled populations, in both periods. K-W, significance of the Kruskal-Wallis test; r_s_, Spearman’s correlation coefficient; * p < 0.050; ** p < 0.010; *** p < 0.001; ns, not significant.

| **Gametes recruitment period (June – September)** | | | |
| --- | --- | --- | --- |
|  |  | **DT (°C)** | **Solar radiation (W/m²)** |
|  | **K-W** | **r_s_** | **r_s_** |
| **Fecundity (#/100 mm^3^)** | ** | 0.500 *** | 0.434 *** |
| **Gonadal Index (%)** | *** | 0.575 *** | 0.518 *** |
| **Diameter (µm)** | *** | 0.086 *** | 0.069 *** |
| **Gametes maturity period (December – March)** | | | |
|  |  | **DT (°C)** | **Solar radiation (W/m²)** |
|  | **K-W** | **rs** | **rs** |
| **Fecundity (#/100 mm3)** | * | 0.254 | 0.101 |
| **Gonadal Index (%)** | ns | - | - |
| **Diameter (µm)** | *** | - 0.109 *** | - 0.017 |
